# Alternative endoscopic salvage therapies using lumen-apposing metal stents for stent misdeployment during endoscopic ultrasound-directed transgastric intervention

**DOI:** 10.1055/a-2422-5789

**Published:** 2024-10-25

**Authors:** Laurent Monino, Lancelot Marique, Yannick Deswysen, Benoit Navez, Etienne Danse, Tom Moreels

**Affiliations:** 1 70492 Hepatogastroenterology, Cliniques universitaires Saint-Luc, Brussels, Belgium; 2 26905 Hepatogastroenterology, Centre Hospitalier Universitaire de Montpellier, Montpellier, France; 3 70492 Surgery, Cliniques universitaires Saint-Luc, Brussels, Belgium; 4 70492 Radiology, Cliniques universitaires Saint-Luc, Brussels, Belgium

The adverse event (AE) rate in endoscopic ultrasound (EUS)-directed transgastric intervention (EDGI) reaches 15% according to the literature 1 2 3 . The most common AE during EDGI is lumen-apposing metal stent (LAMS) misdeployment. Misdeployment types I and II are the most common during EDGI 4 .The stent-in-stent technique using a fully covered metal stent (FCSEMS) seems to be a good salvage option. We report three cases of type II misdeployment during EDGI procedures that were successfully treated without the use of an FCSEMS.

The three patients presented with type II misdeployment during the first step of the EDGI procedure. Endoscopic salvage therapy was performed successfully in all of the cases ( Video 1 ). In one patient the “remove-and-replace” technique was used. The LAMS was removed while the guidewire was secured in the excluded stomach. A new LAMS was then correctly deployed over the wire using the initial gastrogastrostomy fistula ( Fig. 1 ). In the two other patients the “LAMS-in-LAMS” technique was performed. A coaxial LAMS was placed over the guidewire through the misdeployed LAMS. The distal flange was deployed into the excluded stomach and the excluded stomach was pulled against the misdeployed LAMS. The coaxial LAMS was then correctly deployed between the digestive lumens through the misdeployed LAMS ( Fig. 2 ). In all cases, the second step procedure was performed after 2 weeks without any AEs. The LAMS was removed after 1 year for the patient who underwent the remove-and-replace technique. The two LAMSs were removed at 6 months for one of the patients treated with the LAMS-in-LAMS technique, without any AEs or fistula development ( Fig. 3 ).

Two alternative endoscopic salvage therapies using lumen-apposing metal stents (LAMSs) for type II misdeployments during a endoscopic ultrasound-directed transgastric intervention are demonstrated: the “remove-and-replace” and the “LAMS-in-LAMS” techniques.Video 1

**Fig. 1:**
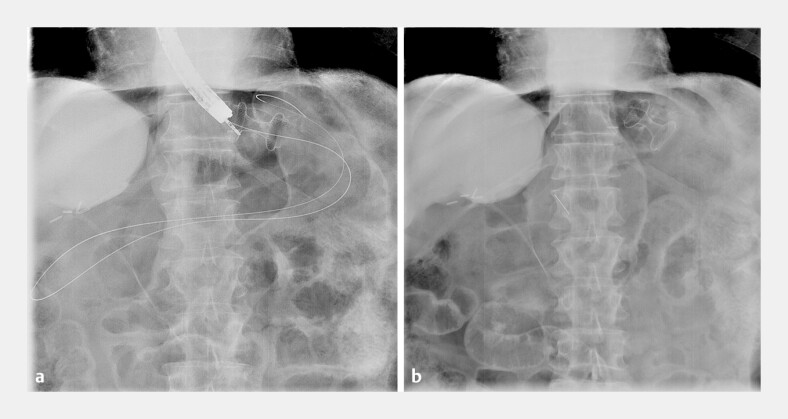
Radiographic images of the remove-and-replace technique being performed over a guidewire showing: **a** removal of the misdeployed lumen-apposing metal stent (LAMS); **b** a new LAMS placed through the initial gastrogastrostomy fistula.

**Fig. 2:**
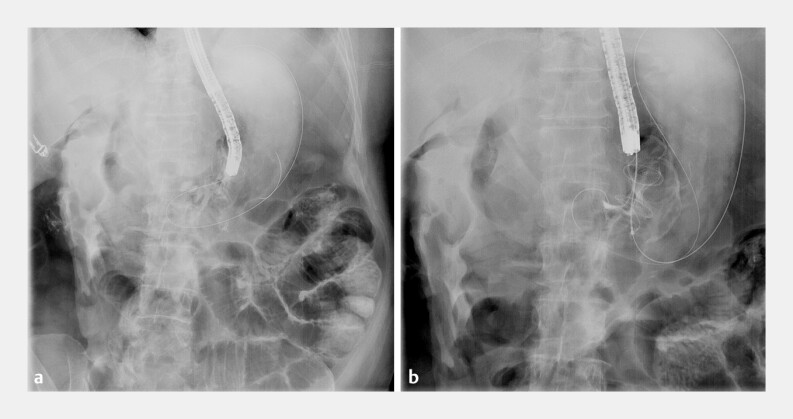
Radiographic images of the lumen-apposing metal stent (LAMS)-in-LAMS technique being performed over a guidewire showing: **a** carboperitoneum confirming the misdeployment; **b** a coaxial LAMS deployed through the misdeployed LAMS.

**Fig. 3:**
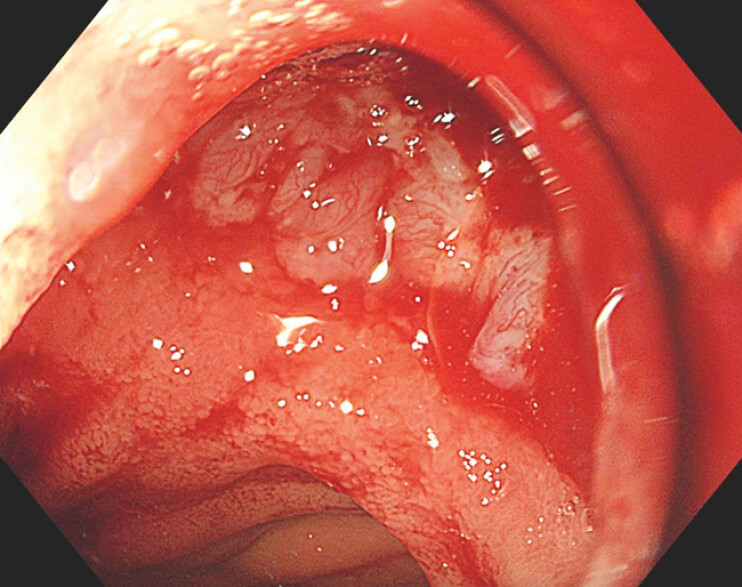
Endoscopic image showing the gastrogastrostomy fistula tract after removal of the lumen-apposing metal stents at 6 months.

The LAMS-in-LAMS technique appears to be easier than the remove-and-replace technique, reducing the number of exchanges and the risk of losing the access maintained by the guidewire. Nevertheless, future studies are needed to confirm that the strength of the anastomosis after the LAMS-in-LAMS technique is at least similar to that in an uncomplicated EDGI procedure.

Endoscopy_UCTN_Code_CPL_1AL_2AB

## Notes

Endoscopy E-Videos https://eref.thieme.de/e-videos
*E-Videos* is an open access online section of the journal *Endoscopy* , reporting on interesting cases and new techniques in gastroenterological endoscopy. All papers include a high-quality video and are published with a Creative Commons CC-BY license. Endoscopy E-Videos qualify for HINARI discounts and waivers and eligibility is automatically checked during the submission process. We grant 100% waivers to articles whose corresponding authors are based in Group A countries and 50% waivers to those who are based in Group B countries as classified by Research4Life (see: https://www.research4life.org/access/eligibility/ ). This section has its own submission website at https://mc.manuscriptcentral.com/e-videos .
